# Cardiac Shock Wave Therapy in Coronary Artery Disease: A Systematic Review and Meta-Analysis

**DOI:** 10.3389/fcvm.2022.932193

**Published:** 2022-07-25

**Authors:** Quan Qiu, Shenjie Chen, Yuangang Qiu, Wei Mao

**Affiliations:** Department of Cardiology, The First Affiliated Hospital of Zhejiang Chinese Medical University, Hangzhou, China

**Keywords:** cardiac shock wave therapy, coronary artery disease, meta-analysis, randomized controlled trials, efficacy

## Abstract

**Objective:**

Coronary artery disease (CAD) has been one of the leading causes of morbidity and mortality worldwide. Cardiac shock wave therapy (CSWT) is a novel and non-invasive therapy for CAD. Therefore, we conducted a systematic review and meta-analysis to evaluate the efficacy of CSWT on CAD.

**Methods and results:**

We performed a comprehensive search of electronic databases such as PubMed, Embase, the Cochrane Library, and Wanfang Data in October 2021. The results were reported as weighted mean difference (WMD) with a 95% confidence interval (CI). Statistical heterogeneity scores were assessed with the standard Cochran’s *Q* test and the *I*^2^ statistic. A total of 8 randomized trials and 2 prospective cohort studies, together involving 643 patients (*n* = 336 CSWT and *n* = 307 control), were included in our study. Eight studies with 371 patients showed significantly improved rest left ventricular ejection fraction (LVEF) with CSWT as compared to that of the control group (WMD 3.88, 95% CI 1.53–6.23, *p* = 0.001, *I*^2^ = 51.2%). Seven studies with 312 patients reported left ventricular internal diameter in diastole (LVIDd) were markedly decreased in the CSWT group compared to the control group (WMD −1.81, 95% CI −3.23 to −0.39, *p* = 0.012, *I*^2^ = 20.3%). The summed stress score significantly favored the CSWT group (WMD −3.76, 95% CI −6.15 to −1.37, *p* = 0.002, *I*^2^ = 56.8%), but there was no significant difference for the summed rest score. Our data were acquired from studies without a perceived high risk of bias, so plausible bias is unlikely to seriously affect the main findings of the current study.

**Conclusion:**

Based on data from our present meta-analysis, CSWT was shown to moderately improve myocardial perfusion and cardiac function among patients with CAD, which would provide the clinicians with a meaningful and valuable option.

**Systematic Review Registration:**

The meta-analysis was registered on the Open Science Framework (OSF) (https://osf.io/r2xf9).

## Introduction

Coronary artery disease (CAD) is one of the most common and severe cardiovascular diseases and causes heavy economic and health burdens globally. CAD has affected nearly 20.1 million people ≥20 years of age in the United States (1). Although optimal medical therapy and revascularization have emerged as effective approaches for CAD treatment, an increased number of patients suffer from chronic angina, which seriously impairs the quality of life (2). Therefore, cardiac shock wave therapy (CSWT) has recently become appealing due to the improvement in angina symptoms.

Emerging evidence have suggested that CSWT, an application of low-intensity shock waves, showed beneficial effects on improvement in angina symptoms and exertional capacity in patients with CAD (3–5). *In vitro* and animal studies indicated that CSWT could exert anti-inflammatory effect, reduce oxidative stress, enhance angiogenesis, inhibit myocardial apoptosis and necroptosis, and regulate autophagy (6–10). However, in clinical studies, investigations into the efficacy of CSWT on cardiac functions and myocardial perfusion have yielded inconsistent and conflicting results. Some studies demonstrated that CSWT could enhance left ventricular (LV) systolic function and alleviate myocardial ischemia (11–13), whereas others found that there were no significant associations between CSWT and cardiac function or myocardial perfusion (14, 15). To address this issue, Burneikaitë et al. (16) and Yang et al. (17) performed meta-analyses in 2017 and 2020, respectively. However, they missed several important studies reported in Chinese, and additional studies have since been published. Moreover, the included studies in their meta-analyses were mainly single-arm studies. Therefore, we performed a meta-analysis to investigate the effect of CSWT on heart functions and myocardial perfusion in CAD based on random placebo-controlled trials.

## Methods

This meta-analysis followed the Preferred Reporting Items for Systematic Reviews and Meta-Analyses (PRISMA) guidelines (Table 1) (18). All the abstracts were screened by two independent methodologically trained reviewers (QQ and SJC). Discrepancies were resolved by discussion between the two researchers, if necessary, by a third reviewer (YGQ). The full-texts screening was evaluated in a similar manner to abstract screening. We used EndNote X8 (Clarivate, Pennsylvania, PA, United States) as literature management software for potentially eligible studies in the selection process.

**TABLE 1:** PRISMA table.

Section and topic	Item #	Checklist item	Location where item is reported
Title			
Title	1	Identify the report as a systematic review.	p1
Abstract			
Abstract	2	See the PRISMA 2020 for Abstracts checklist.	Table 1
Introduction			
Rationale	3	Describe the rationale for the review in the context of existing knowledge.	p2
Objectives	4	Provide an explicit statement of the objective(s) or question(s) the review addresses.	p2
Methods			
Eligibility criteria	5	Specify the inclusion and exclusion criteria for the review and how studies were grouped for the syntheses.	p3, 4
Information sources	6	Specify all databases, registers, websites, organizations, reference lists and other sources searched or consulted to identify studies. Specify the date when each source was last searched or consulted.	p2–3
Search strategy	7	Present the full search strategies for all databases, registers and websites, including any filters and limits used.	p2 and Supplementary Material 1
Selection process	8	Specify the methods used to decide whether a study met the inclusion criteria of the review, including how many reviewers screened each record and each report retrieved, whether they worked independently, and if applicable, details of automation tools used in the process.	p2
Data collection process	9	Specify the methods used to collect data from reports, including how many reviewers collected data from each report, whether they worked independently, any processes for obtaining or confirming data from study investigators, and if applicable, details of automation tools used in the process.	p3
Data items	10a	List and define all outcomes for which data were sought. Specify whether all results that were compatible with each outcome domain in each study were sought (e.g., for all measures, time points, analyses), and if not, the methods used to decide which results to collect.	Table 3
	10b	List and define all other variables for which data were sought (e.g., participant and intervention characteristics, funding sources). Describe any assumptions made about any missing or unclear information.	Table 2
Study risk of bias assessment	11	Specify the methods used to assess risk of bias in the included studies, including details of the tool(s) used, how many reviewers assessed each study and whether they worked independently, and if applicable, details of automation tools used in the process.	p3
Effect measures	12	Specify for each outcome the effect measure(s) (e.g., risk ratio, mean difference) used in the synthesis or presentation of results.	p3
Synthesis methods	13a	Describe the processes used to decide which studies were eligible for each synthesis [e.g., tabulating the study intervention characteristics and comparing against the planned groups for each synthesis (item #5)].	p3–4
	13b	Describe any methods used to synthesize results and provide a rationale for the choice(s). If meta-analysis was performed, describe the model(s), method(s) to identify the presence and extent of statistical heterogeneity, and software package(s) used.	p3–4
	13c	Describe any sensitivity analyses conducted to assess robustness of the synthesized results.	p5–6
Reporting bias assessment	14	Describe any methods used to assess risk of bias due to missing results in a synthesis (arising from reporting biases).	–
Certainty assessment	15	Describe any methods used to assess certainty (or confidence) in the body of evidence for an outcome.	–
Results			
Study selection	16a	Describe the results of the search and selection process, from the number of records identified in the search to the number of studies included in the review, ideally using a flow diagram.	p4 and Figure 1
	16b	Cite studies that might appear to meet the inclusion criteria, but which were excluded, and explain why they were excluded.	p4
Study characteristics	17	Cite each included study and present its characteristics.	Table 3
Risk of bias in studies	18	Present assessments of risk of bias for each included study.	Supplementary Material 2
Results of individual studies	19	For all outcomes, present, for each study: (a) summary statistics for each group (where appropriate) and (b) an effect estimate and its precision (e.g., confidence/credible interval), ideally using structured tables or plots.	Table 3
Results of syntheses	20a	For each synthesis, briefly summarize the characteristics and risk of bias among contributing studies.	p5
	20b	Present results of all statistical syntheses conducted. If meta-analysis was done, present for each the summary estimate and its precision (e.g., confidence/credible interval) and measures of statistical heterogeneity. If comparing groups, describe the direction of the effect.	p5
	20c	Present results of all investigations of possible causes of heterogeneity among study results.	p5, 7, 8
	20d	Present results of all sensitivity analyses conducted to assess the robustness of the synthesized results.	p6
Reporting biases	21	Present assessments of risk of bias due to missing results (arising from reporting biases) for each synthesis assessed.	–
Certainty of evidence	22	Present assessments of certainty (or confidence) in the body of evidence for each outcome assessed.	p5
Discussion			
Discussion	23a	Provide a general interpretation of the results in the context of other evidence.	p6
	23b	Discuss any limitations of the evidence included in the review.	p8
	23c	Discuss any limitations of the review processes used.	p8
	23d	Discuss implications of the results for practice, policy, and future research.	p8
Other information			
Registration and protocol	24a	Provide registration information for the review, including register name and registration number, or state that the review was not registered.	p1
	24b	Indicate where the review protocol can be accessed, or state that a protocol was not prepared.	–
	24c	Describe and explain any amendments to information provided at registration or in the protocol.	–
Support	25	Describe sources of financial or non-financial support for the review, and the role of the funders or sponsors in the review.	p8
Competing interests	26	Declare any competing interests of review authors.	p8
Availability of data, code and other materials	27	Report which of the following are publicly available and where they can be found: template data collection forms; data extracted from included studies; data used for all analyses; analytic code; any other materials used in the review.	–
			

**TABLE 2:** The CSWT operation protocol of the included studies.

	Therapy regimen	Frequency and energy	Location	Device
Èelutkienë et al. (21)	9 sessions with 3 sessions per week; the first, fifth, and the ninth study weeks; 3-month period; 12 spots/session	100 impulses/spot	Whole LV	Cardiospec Medispec, Germantown, MD, United States
Weijing et al. (22)	Thrice weekly (first, third, and fifth days); the first, fifth, and the ninth study weeks; 3-month period; 9 spots/session	200 impulses/spot; 0.09 mJ/mm^2^	Target ischemic session	Modulith SLC; Storz Medical, Switzerland
Jia et al. (11)	Thrice weekly (first, third, and fifth days); the first, fifth, and the ninth study weeks; 3-month period; 9 spots/session	200 impulses/spot; 0.09 mJ/mm^2^	Target ischemic sessions	Modulith SLC; Storz Medical, Switzerland
Mengxian et al. (26)	Thrice weekly (first, third, and fifth days); the first, fifth, and the ninth study weeks; 3-month period; 9 spots/session	200 impulses/spot; 0.09 mJ/mm^2^	Target ischemic sessions	Modulith SLC; Storz Medical, Switzerland
Kagaya et al. (23)	Second, fourth, and sixth days since AMI; 3 sessions in the ischemic border zone around the infarcted myocardium; 9 spots/session/day	200 impulses/spot; 0.09 mJ/mm^2^	Ischemic border zone around the infarcted area	Modulith SLC; Storz Medical, Switzerland
Alunni et al. (24)	The first, fifth, and the ninth study weeks; 3-month period; 10 spots/session	100 impulses/spot; 0.09 mJ/mm^2^	3 target sessions in the ischemic zone	Cardiospec Medispec, Germantown, MD, United States
Wang et al. (25)	Thrice weekly (first, third, and fifth days); first, fifth, and ninth study weeks; 3-month period; 9 spots/session	200 impulses/spot; 0.09 mJ/mm^2^	Target ischemic session	Modulith SLC; Storz Medical, Switzerland
Wang et al. (25)	Thrice weekly (first, third, and fifth days); 1-month period; 9 spots/session	200 impulses/spot; 0.09 mJ/mm^2^	Target ischemic session	Modulith SLC; Storz Medical, Switzerland
Zhang et al. (28)	Thrice weekly (first, third, and fifth days); 1-month period; 9 spots/session	200 impulses/spot; 0.09 mJ/mm^2^	Target ischemic session	Modulith SLC; Storz Medical, Switzerland
Lan et al. (27)	Thrice weekly (first, third, and fifth days); 1-month period; 9 spots/session	200 impulses/spot; 0.09 mJ/mm^2^	Target ischemic session	Modulith SLC; Storz Medical, Switzerland
Peng et al. (29)	Thrice weekly (first, third, and fifth days); first, fifth, and ninth study weeks; 3-month period; 9 spots/session	200 impulses/spot; 0.09 mJ/mm^2^	Target ischemic session	Modulith SLC; Storz Medical, Switzerland

**TABLE 3:** Characteristics of the included studies.

Study	Year	Trial type	Study population	Region	Age (mean)	M/F	Follow-up (m)	LVEF baseline (%)	Myocardial perfusion	CSWT/Con	Randomized methods	Control group
Èelutkienë et al. (21)	2019	RCT	Stable angina	Lithuania	67.2 ± 7.8/69.4 ± 7.8	45/14	6	46.5 ± 10.6/48.5 ± 9.0	8.5 (5.3; 12.8)/10.0 (4.0; 15.0)	30/29	Random number table	Sham procedure
Weijing et al. (22)	2021	RCT	Refractory angina	China	68.1 ± 6.7/68.9 ± 6.6	61/26	6	–	16.27 ± 7.64/16.45 ± 5.05	46/41	NR	Medical therapy
Jia et al. (11)	2021	RCT	Severe CAD	China	69.20 ± 11.33/71.40 ± 9.71	21/9	3	62.5 (60, 65)/62.5 (60, 65)	17.63 ± 7.86/11.23 ± 5.69	15/15	Random number table	Sham procedure
Mengxian et al. (26)	2012	RCT	Severe CAD	China	63.71 ± 8.60/66.45 ± 8.51	18/7	6	51.36 ± 4.27/50.18 ± 4.55	–	14/11	NR	Sham Procedure
Kagaya et al. (23)	2017	Cohort study	MI	Japan	65.0 ± 7.3/67.3 ± 12.8	27/5	12	58.7 ± 8.2/54.4 ± 12.3	–	17/25	NR	NR
Alunni et al. (24)	2015	Prospective cohort study	Refractory angina	Italy	70 ± 9.5/71 ± 5.3	63/9	6	56.4 ± 10.3/57.3 ± 9.6	–	43/29	NR	NR
Wang et al. (25)	2010	RCT	End-stage CAD	China	63 ± 10/69 ± 7	30/5	3	53.1 ± 12.8/54.3 ± 13.9	–	16/10	NR	NR
Wang et al. (25)	2010	RCT	End-stage CAD	China	63 ± 10/69 ± 7	30/5	1	56.1 ± 13.2/54.3 ± 13.9	–	9/10	NR	NR
Zhang et al. (28)	2021	RCT	RA	China	65.83 ± 6.3/64.4 ± 6.7	53/17	6	50.32 + 12.69/50.21 ± 10.01	320.10 ± 3.45/30.28 ± 2.34	38/32	NR	Medical therapy
Lan et al. (27)	2016	RCT	Ischemic HF	China	67 ± 6/66 ± 7	39/14	3	37.41 ± 5.87/38.31 ± 4.56	21.46 ± 9.51/23.58 ± 7.52	28/25	NR	Sham procedure
Peng et al. (29)	2018	RCT	Ischemic HF	China	62.5 ± 6.8/61.3 ± 7.2	100/80	3	44.40 ± 6.32/44.12 ± 12.52	22.91 ± 4.32/22.05 ± 4.07	90/90	NR	Sham procedure
												

**FIGURE 1:**
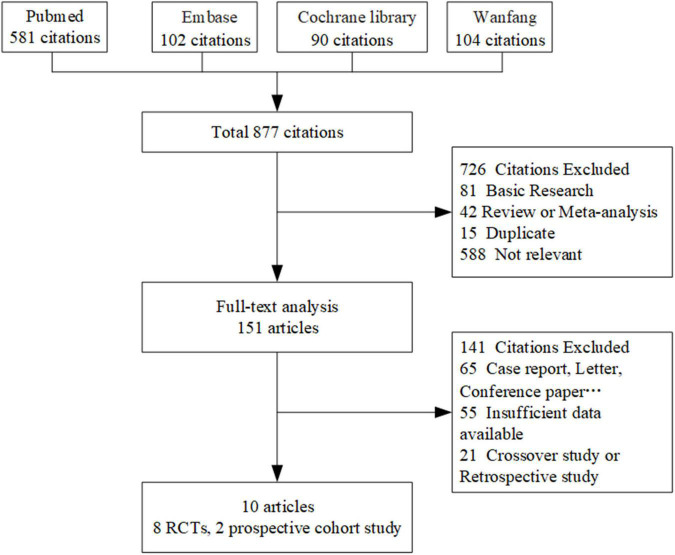
Flow chart of the included studies. RCTs indicates randomized controlled trials.

### Literature Search

A comprehensive systematic search strategy (Supplementary Material 1) was developed to retrieve relevant articles. Our objective was to identify all the randomized controlled trials (RCTs) and prospective cohort studies comparing the effect of CSWT on cardiac functions and myocardial perfusion with a placebo. PubMed, the Cochrane Library, Embase, and Wanfang Data were searched from January 1999 to November 2021 for English and Chinese language publications. Medical subject headings (MeSH terms) and keywords included cardiovascular disease, CAD, and angina pectoris combined with extracorporeal shockwave therapy.

### Study Selection

We included studies in this meta-analysis fulfilling the following criteria: (1) randomized trials or prospective cohort studies; (2) studies involving patients with CAD confirmed by coronary angiography or CT angiography; (3) studies on the use of CSWT as the intervention; and (4) studies presenting all outcomes of interest.

Studies were excluded from the analysis if they were (1) basic science studies, case reports, letters, conference proceedings, reviews, or duplicated publications; (2) publications that did not report any outcomes of interest; (3) studies that lacked the placebo groups; and (4) trials investigating the efficacy of CSWT combination with stem cell therapy.

### Study Quality Assessment and Risk of Bias

Two reviewers (QQ and YGQ) independently performed the quality evaluation. Assessment of the risk of bias in each included randomized trial was performed in accordance with the revised Cochrane risk-of-bias tool (RoB 2) (19). For cohort studies, we used the Risk of Bias in Non-randomized Studies of Interventions (ROBINS-I) tool to assess the risk of bias (20). The risk of bias was evaluated in domains, including confounding, selection of participants into the study, classification of interventions, deviation from intended intervention, missing outcome data, measurement of the outcome, and selection of the reported result.

### Data Extraction and Outcome Measures

Completed data from each study were extracted independently by two of the authors (QQ and SJC) using a standardized data extraction sheet. We extracted relevant information, including the first author, year of publication, trial design, trial duration, treatment regimen, patients’ information, and characteristics of an outcome. Our primary outcome was global LV function and myocardial perfusion.

### Statistical Analysis

We presented continuous data with normal distribution as mean value ± SD and non-normal data as median with interquartile range (IQR) (Q1, Q3). We analyzed results from randomized trials or prospective cohort studies that had placebo controls. We summarized all the continuous outcome data using weighted mean differences (WMDs) and their 95% confidence intervals (CIs). Heterogeneity was assessed using Cochran’s *Q* test and expressed by *I*^2^ statistic. If *I*^2^ ≥ 50% or the *p*-value < 0.05 for the *Q*-statistic, it indicated significant heterogeneity. The random-effects models were used in the presence of heterogeneity, and if there was no heterogeneity among studies, the fixed-effects models were performed. Publication bias was assessed by drawing a funnel plot and tested with Egger’s test. Statistical analyses were performed using STATA 15 (StataCorp LP, College Station, TX, United States).

## Results

### Study Characteristics and Patient Population

By searching 4 databases, 877 eligible publications involving CSWT were identified and 726 records were initially excluded after screening the title and the abstract. Then, 151 records were included for a more thorough review using the inclusion and exclusion criteria described in the methods. Finally, 10 records (11, 21–29) were selected for review following the PRISMA statement. Among them, eight were RCTs and two were prospective cohort studies. The number of publications due to reasons for exclusion at each stage of the eligibility assessment is given in Figure 1. Several prospective placebo-controlled cohort studies and RCTs, which evaluated the effect of CSWT on relieving symptoms in patients with angina pectoris or CAD, were excluded from our study for lack of inadequate data on echocardiography or myocardial perfusion after full-text screening (30–32). In Yang’s study (33), their data could not combine with others; therefore, we excluded it.

In total, 643 participants were included in this study with 336 cases treated with CSWT. Two publications were performed in Europe (Lithuania and Italy) and eight publications were performed in Asia (China and Japan) and published between 2010 and 2021. Investigators used CSWT as an alternative treatment option for stable angina, refractory angina, severe CAD, end-stage CAD, ischemic heart failure, and acute myocardial infarction (AMI). The reported mean or median age for studies were ranging from 56.6 to 71.4 years and 30.8% were women. The most commonly used CSWT operation protocols were the high-frequency treatment regimen completing nine CSWTs in 3 weeks and a low-frequency treatment regimen in which CSWT was performed three times weekly during the first week of each month within 3 months. Table 2 describes the detailed CSWT protocols of included studies. The follow-up time of the studies ranged from 1 to 12 months. The common characteristics and the CSWT operation protocols of included studies are given in Tables 2, 3, respectively.

### Risk of Bias

Based on the methodological quality assessment (Supplementary Material 2), six studies were considered as having a moderate risk of bias and four studies were assessed as having a low risk of bias.

### Meta-Analysis of Cardiac Shock Wave Therapy Effect on Left Ventricular Function

**FIGURE 2:**
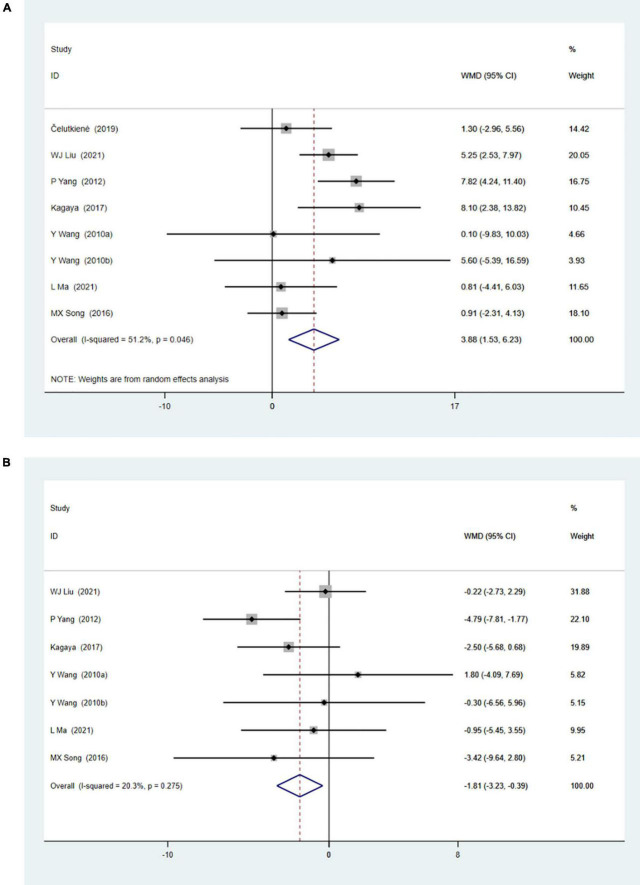
**(A)** Forrest map of overall impact of cardiac shock wave therapy on LVEF (WMD 3.88, 95% CI 1.53–6.23, *p* = 0.001). The *I*^2^ value revealed considerable heterogeneity across studies (*I*^2^ = 51.2%; *p* = 0.046). **(B)** Forrest map of overall impact of cardiac shock wave therapy on LVIDd (WMD –1.81, 95% CI –3.23 to –0.39, *p* = 0.012). The *I*^2^ value revealed considerable heterogeneity across studies (*I*^2^ = 20.3%; *p* = 0.275).

Eight studies with 371 patients reported changes of rest left ventricular ejection fraction (LVEF) by echocardiography. Meta-analysis showed significant improvement of rest LVEF due to CSWT (WMD 3.88, 95% CI 1.53–6.23, *p* = 0.001, *I*^2^ = 51.2%), as seen in Figure 2A. Seven studies with 312 patients reported left ventricular internal diameter in diastole (LVIDd) data, and the result (Figure 2B) showed decreased LVIDd when comparing the CSWT group to the control group (WMD −1.81, 95% CI −3.23 to −0.39, *p* = 0.012, *I*^2^ = 20.3%).

### Meta-Analysis of Cardiac Shock Wave Therapy Effect on Myocardial Perfusion

**FIGURE 3:**
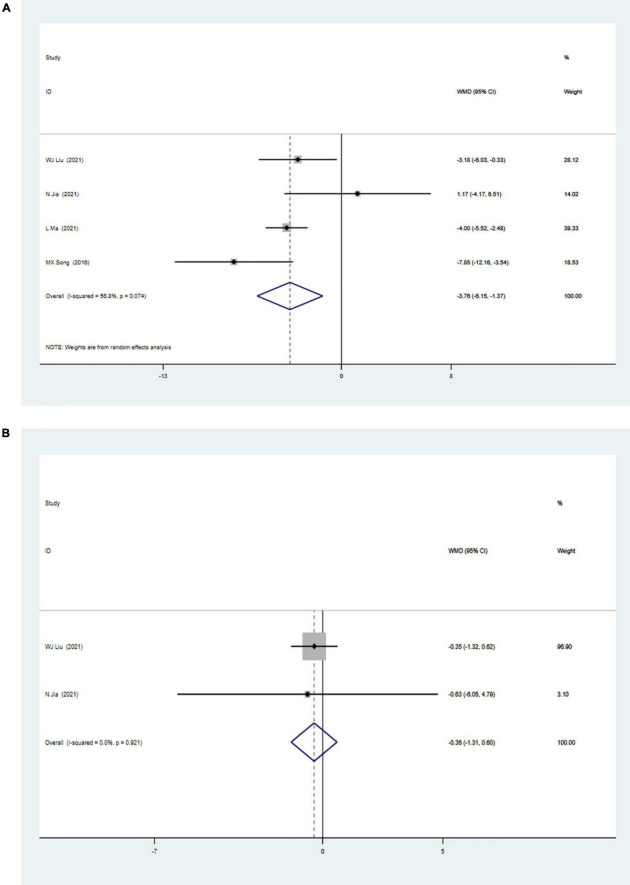
**(A)** Forrest map of overall impact of cardiac shock wave therapy on SSS (WMD –3.76, 95% CI –6.15 to –1.37, *p* = 0.002). The *I*^2^ value revealed considerable heterogeneity across studies (*I*^2^ = 56.8%; *p* = 0.074). **(B)** Forrest map of overall impact of cardiac shock wave therapy on SRS (WMD –0.36, 95% CI –1.31 to 0.60, *p* = 0.462). The *I*^2^ value revealed considerable heterogeneity across studies (*I*^2^ = 0.0%; *p* = 0.021).

The meta-analysis of myocardial perfusion was based on the comparison between CSWT and placebo (control) on the parameters of the summed stress score (SSS) and the summed rest score (SRS) detected by single-photon emission CT. Four studies with 231 patients were included in the analysis of the effect of CSWT on the SSS, and the result (Figure 3A) of our meta-analysis showed significant improvement of the SSS in the CSWT group compared with placebo (WMD −3.76, 95% CI −6.15 to −1.37, *p* = 0.002, *I*^2^ = 56.8%). Only Liu and Jia reported the SRS data (Figure 3B), and there was no significant difference between the two groups (WMD −0.36, 95% CI −1.31 to 0.60, *p* = 0.462, *I*^2^ = 0.0%).

### Publication Bias

**FIGURE 4:**
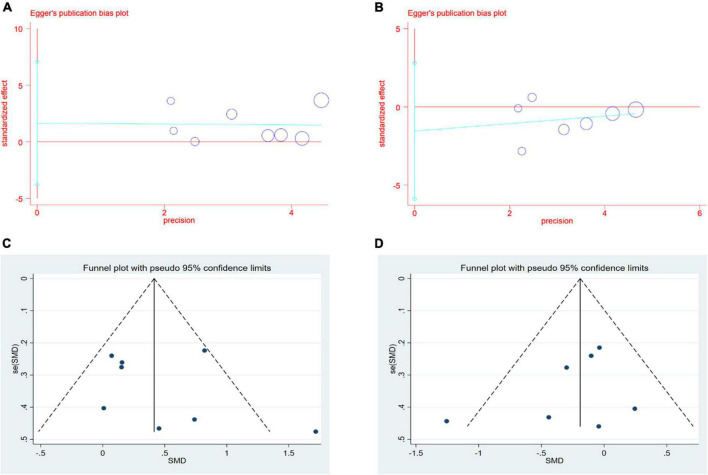
**(A,B)** Results of Egger’s test for LVEF and LVIDd. **(C,D)** Results of Funnel plot for LVEF and LVIDd.

Formal investigation using funnel plot and Egger’s test revealed no publication bias in the meta-analyses for the effect of CSWT on LVEF and LVIDd (Figures 4A–D). The Egger’s test results of LVEF and LVIDd showed *p*-values of 0.48 and 0.36, respectively (Figures 4A,B). The asymmetry of a funnel plot may result from different baseline characteristics of participants and from differences in the medical treatment of patients with CAD in all the studies (Figures 4C,D). We did not test publication bias for the meta-analyses of CSWT effect on the SSS and the SRS because too few studies were available to make a valid statistical test.

### Sensitivity Analysis

**FIGURE 5:**
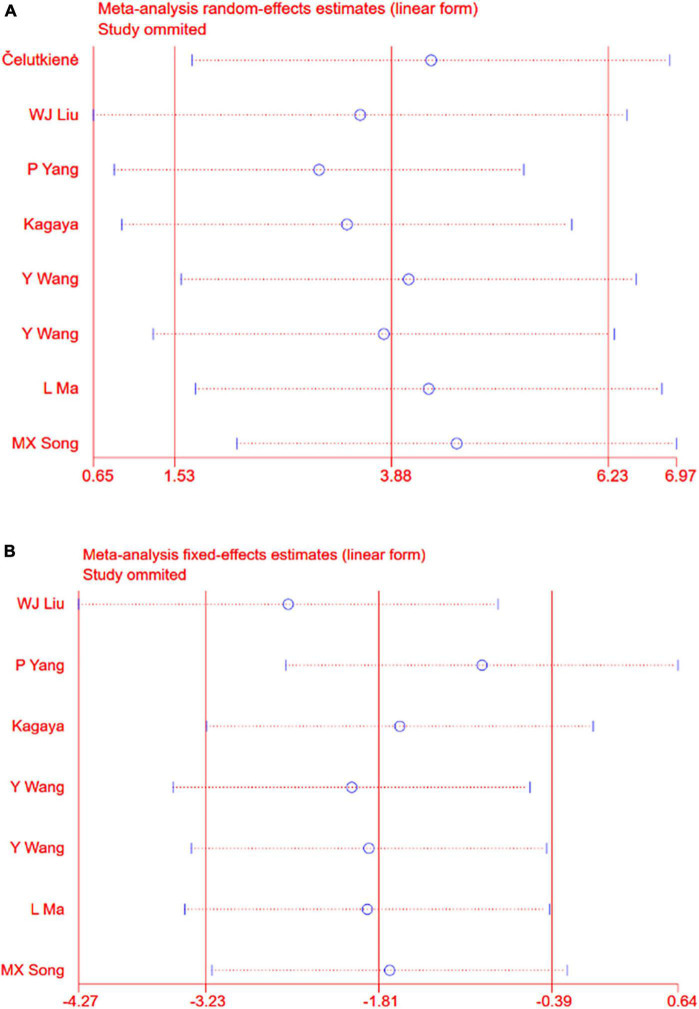
**(A)** Sensitivity analysis for the LVEF. **(B)** Sensitivity analysis for the LVIDd.

**FIGURE 6:**
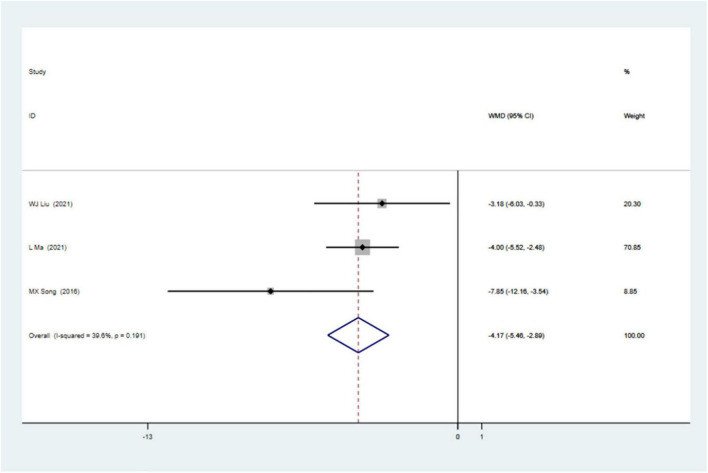
Forrest map of updated impact of cardiac shock wave therapy on SSS (WMD –4.17, 95% CI –5.46 to –2.89, *p* < 0.001). The *I*^2^ value revealed considerable heterogeneity across studies (*I*^2^ = 39.6%; *p* = 0.191).

Sensitivity analyses removing one study at a time revealed that the size and the direction of the pooled estimates of the effect of CSWT on LVEF and LVIDd were consistent for all the results (Figures 5A,B). Because there was a significant difference between the two groups on the SSS at baseline in one study, we excluded it and still found a significant difference comparing the CSWT group with placebo without significant heterogeneity (WMD −4.17, 95% CI −5.46 to −2.89, *p* < 0.001, *I*^2^ = 39.6%) (Figure 6).

## Discussion

The inconsistent conclusions about the effect of CSWT on cardiac function call for more rigorous studies to demonstrate the efficacy of CSWT for patients with CAD. By incorporating eight RCTs and two prospective cohort studies, our meta-analysis provided a relatively higher quality of evidence to increase the assurance of administering CSWT to patients with CAD. It is important to note that CSWT could moderately improve myocardial function and prevent ventricular remodeling (supported by remarkably improved LVEF and decreased LVIDd). Besides, the analysis also revealed that CSWT may improve myocardial perfusion (supported by the decrease of the SSS).

The CSWT technology has proven to be safe in more than 60 medical centers worldwide. Accumulating evidence has demonstrated that CSWT does not cause an increase in the levels of cardiac biomarkers (i.e., troponin I, troponin T, creatine kinase-MB, B-type natriuretic peptide) and has no adverse effects on blood pressure, heart rate, and oxygen saturation in patients with CAD. Serious complications, including malignant arrhythmia and embolism, have not been observed after CSWT (11). In Kagaya’s study (23), which enrolled 17 patients with AMI undergoing CSWT, 16 patients completed CSWT and survived 12 months after AMI without any adverse effects with only one patient dying of cardiac rupture independent of CSWT. In Ceccon’s study (34), 15 patients with refractory angina receiving CSWT presented mild chest discomfort during treatment and with a rare appearance of major adverse effects related to CSWT. In Jia’s study (11), 15 patients diagnosed with severe CAD were treated with CSWT and no severe adverse effects occurred. Since shock wave carries low-intensity energy, there are several contradictions to CSWT, including acute pericarditis, acute myocarditis, infectious endocarditis, deep venous thromboembolism, intracardiac thrombus, severe aortic valve stenosis, thoracic aortic aneurysm, thoracic aortic dissecting aneurysm, cardiac transplantation, pulmonary embolism, and mechanical heart valve replacement (11, 21).

Angina is the most common symptom of coronary heart disease, and it has been indicated that the mortality rate of refractory angina is 3–4% per year (35). Angina is one of the main indications of CSWT. CSWT generator produces pulse waves that could propagate through myocardial tissues and vascular endothelial cell membranes (6, 36). Many clinical trials have shown that CSWT could alleviate angina symptoms as assessed by the improved Seattle Angina Questionnaire score and the Canadian Cardiovascular Society grading of the angina pectoris, a decreased nitroglycerin dosage, and improved exercise tolerance assessed by increased 6-min walk distance (3, 11, 30).

Porcine, mice, or rat AMI or ischemic heart failure models were used in previous studies to demonstrate the improvement of LVEF or wall thickening fraction (6, 9, 37). At present, some studies indicated that CSWT could effectively improve myocardial function and perfusion in patients with stable angina and severe CAD at rest and during stress (11, 14, 21, 38, 39). Yang et al. (17) showed that CSWT significantly improved LVEF compared to baseline in their meta-analysis based on six single-arm studies and one cohort study, while the improvement may be due to optimal medical therapy and lifestyle modifications. Our study based on RCTs minimized various confounders and selection bias and had a higher level of statistical reliability. A recent meta-analysis of CSWT for stable CAD also reported that CSWT could improve LVEF (16). However, it should be noted that this study included single-arm, non-randomized, and randomized trials and they did not perform statistical analysis. Because RCTs are considered the gold standard evidence for determining the efficacy of interventions and our meta-analysis is based mainly on RCTs, our data have good internal validity.

There are two brands of CSWT machines used in the included studies. Both of the machines worked in a similar way to deliver shockwaves to the target ischemic zones in an R-wave-triggered manner. There were few head-to-head trials to compare the efficiency of the two different brands of machines. However, it seems that there is not much difference between the two kinds of machines. In this meta-analysis, Alunni’s and Celutkiene’s study (21, 24) chose the Cardiospec Medispec CSWT machine but the other studies used the Modulith SLC machine. Moreover, the included clinical trials did not share a uniform treatment protocol. Most of the studies included in the meta-analysis received two different courses of CSWT treatment, including a 1-month course (25–27) and a 3-month course (11, 21, 22, 24, 25). A total of 100 or 200 impulses were delivered to the target area with an energy flux density of 0.09 mJ/mm^2^. Other parameters, such as location and frequency, differed subtly among these studies. We have acknowledged this as a limitation of the current meta-analysis, and future studies are needed to be performed according to the standard protocols.

### Limitation

There are some limitations to this study. First, this meta-analysis is limited owing to a lack of large-scale RCTs and the long-term effect of CSWT on CAD. For example, Zhang et al. (28) conducted the largest RCT consisting of 180 participants and performed echocardiography evaluation before and 3 months after CSWT treatment. Time effect should be considered when evaluating the clinical outcomes of CSWT. Second, the sonographers who performed echocardiography affected the accuracy and precision of LVEF and LVIDd measurements, and this interpersonal variability had been shown to exist even when echocardiographic image acquisition was performed according to echocardiography guidelines. Moreover, it seemed possible that, due to sparse RCTs, this meta-analysis included all the known studies, and over half of the studies were conducted in Asia, which might influence the representativeness of the population.

## Conclusion

Taken together, the present meta-analysis of these studies showed that CSWT appeared to be effective in improving myocardial perfusion and cardiac function in patients with CAD. CSWT is a promising therapeutic modality for the treatment of CAD. More high-quality RCTs with large samples and long-term follow-up are urgently needed to further confirm our results.

## Data Availability Statement

The original contributions presented in this study are included in the article/Supplementary Material, further inquiries can be directed to the corresponding author.

## Author Contributions

WM designed the study. QQ and SC screened and evaluated the studies and performed a comprehensive characterization of the studies. QQ performed the statistical analyses. YQ checked the included studies. YQ and SC checked the statistical analyses. QQ and WM wrote the manuscript. All authors have contributed to the article and approved the submitted version of the manuscript.

## Conflict of Interest

The authors declare that the research was conducted in the absence of any commercial or financial relationships that could be construed as a potential conflict of interest.

## Publisher’s Note

All claims expressed in this article are solely those of the authors and do not necessarily represent those of their affiliated organizations, or those of the publisher, the editors and the reviewers. Any product that may be evaluated in this article, or claim that may be made by its manufacturer, is not guaranteed or endorsed by the publisher.
