# Supplementary material for: Cardiac Shock Wave Therapy in Coronary Artery Disease: A Systematic Review and Meta-Analysis
Source: Front Cardiovasc Med. 2022 Jul 25;9:932193. doi: 10.3389/fcvm.2022.932193 (PMC9358011; doi:10.3389/fcvm.2022.932193)
Supplement: Supplementary file 1 [file Table_1.DOCX]

Literature search strategy for all the databases

**1. Search strategy for Pubmed**

# 1 Extracorporeal Shockwave Therapy [Mesh]

# 2 ((Extracorporeal Shock Wave Therapy) or (Therapy, Extracorporeal Shockwave) or (Shockwave Therapies, Extracorporeal) or (Shockwave Therapy, Extracorporeal) or (Extracorporeal Shockwave Therapies) or (Therapy, Shock Wave) or (Shock Wave Therapy) or (Shock Wave Therapies))

# 3 Angina Pectoris [Mesh]

# 4 Angina or Pectoris or Stenocardia or Stenocardias or Angor Pectoris

# 5 Coronary Artery Disease [Mesh]

# 6 ((Artery Disease, Coronary) or (Artery Diseases, Coronary) or (Coronary Artery Diseases) or (Left Main Coronary Artery Disease) or (Left Main Disease) or (Left Main Diseases) or (Left Main Coronary Disease) or (Coronary Arteriosclerosis) or (Arterioscleroses, Coronary) or (Coronary Arterioscleroses) or (Atherosclerosis, Coronary) or (Atheroscleroses, Coronary) or (Coronary Atheroscleroses) or (Coronary Atherosclerosis) or (Arteriosclerosis, Coronary))

# 7 Cardiovascular Disease [Mesh]

# 8 ((Diseases, Cardiovascular) or (Disease, Cardiovascular) or (Cardiovascular Diseases))

# 9 (#1 or #2) and (#3 or #4 or #5 or #6 or #7 or #8) Filters: Abstract

**2. Search strategy for Embase**

# 1 'shock wave therapy'/exp 2,225

# 2 'shock wave therapy'/exp OR 'shock wave therapy' OR (('shock'/exp OR shock) AND ('wave'/exp OR wave) AND ('therapy'/exp OR therapy)) 20,037

# 3 'angina pectoris'/exp 110,508

# 4 'angina pectoris'/exp OR 'angina pectoris' OR (('angina'/exp OR angina) AND pectoris) 115,733

# 5 'coronary artery disease'/exp 381,878

# 6 'coronary artery disease'/exp OR 'coronary artery disease' OR (coronary AND ('artery'/exp OR artery) AND ('disease'/exp OR disease)) 596,458

# 7 'cardiovascular disease'/exp 5,008,027

# 8 'cardiovascular disease'/exp OR 'cardiovascular disease' OR (('cardiovascular'/exp OR cardiovascular) AND ('disease'/exp OR disease)) 5,338,033

#9 #1 OR #2 20,037

#10 #3 OR #4 115,733

#11 #5 OR #6 596,458

#12 #7 OR #8 5,338,033

#13 #9 AND #10 295

#14 #9 AND #11 959

#15 #9 AND #12 4,393

#16 #15 AND ('shock wave generator'/dv OR 'shock wave lithotripter'/dv) AND ('animal cell'/de OR 'animal experiment'/de OR 'animal model'/de OR 'animal tissue'/de OR 'case control study'/de OR 'case report'/de OR 'case study'/de OR 'clinical article'/de OR 'clinical study'/de OR 'clinical trial'/de OR 'clinical trial protocol'/de OR 'clinical trial topic'/de OR 'cohort analysis'/de OR 'comparative effectiveness'/de OR 'comparative study'/de OR 'control group'/de OR 'controlled clinical trial'/de OR 'controlled study'/de OR 'diagnostic test accuracy study'/de OR 'double blind procedure'/de OR 'evidence based medicine'/de OR 'ex vivo study'/de OR 'experimental model'/de OR 'feasibility study'/de OR 'human'/de OR 'human cell'/de OR 'human tissue'/de OR 'in vitro study'/de OR 'in vivo study'/de OR 'intermethod comparison'/de OR 'intervention study'/de OR 'longitudinal study'/de OR 'major clinical study'/de OR 'medical record review'/de OR 'meta analysis'/de OR 'multicenter study'/de OR 'multicenter study topic'/de OR 'non inferiority trial'/de OR 'nonhuman'/de OR 'observational study'/de OR 'pilot study'/de OR 'practice guideline'/de OR 'prospective study'/de OR 'questionnaire'/de OR 'randomized controlled trial'/de OR 'randomized controlled trial topic'/de OR 'single blind procedure'/de) AND ([adult]/lim OR [aged]/lim OR [middle aged]/lim OR [very elderly]/lim OR [young adult]/lim) AND ('acute coronary syndrome'/dm OR 'angina pectoris'/dm OR 'artery calcification'/dm OR 'artery lesion'/dm OR 'bleeding'/dm OR 'cardiovascular disease'/dm OR 'cerebrovascular accident'/dm OR 'chronic kidney failure'/dm OR 'chronic total occlusion'/dm OR 'claudication'/dm OR 'complication'/dm OR 'coronary artery calcification'/dm OR 'coronary artery disease'/dm OR 'coronary artery obstruction'/dm OR 'coronary artery occlusion'/dm OR 'diabetes mellitus'/dm OR 'diabetic nephropathy'/dm OR 'dyslipidemia'/dm OR 'dyspnea'/dm OR 'extravasation'/dm OR 'false aneurysm'/dm OR 'fever'/dm OR 'heart arrhythmia'/dm OR 'heart infarction'/dm OR 'heart muscle ischemia'/dm OR 'hematoma'/dm OR 'hyperlipidemia'/dm OR 'hypertension'/dm OR 'hypotension'/dm OR 'in-stent restenosis'/dm OR 'mitral valve regurgitation'/dm OR 'non insulin dependent diabetes mellitus'/dm OR 'non st segment elevation myocardial infarction'/dm OR 'obesity'/dm OR 'pain'/dm OR 'peripheral occlusive artery disease'/dm OR 'pneumothorax'/dm OR 'postoperative complication'/dm OR 'recurrent disease'/dm OR 'restenosis'/dm OR 'spasticity'/dm OR 'st segment elevation myocardial infarction'/dm OR 'stent underexpansion'/dm OR 'thorax pain'/dm) 102

**3. Search strategy for Cochrane**

#1 MeSH descriptor: [Extracorporeal Shockwave Therapy] explode all trees 149

#2 "Extracorporeal Shock Wave Therapy" or "Therapy, Extracorporeal Shockwave" or "Shockwave Therapies, Extracorporeal" or "Shockwave Therapy, Extracorporeal" or "Extracorporeal Shockwave Therapies" or "Therapy, Shock Wave" or "Shock Wave Therapy" or "Shock Wave Therapies" 1076

#3 #1 or #2 1092

#4 MeSH descriptor: [Angina Pectoris] explode all trees 4665

#5 "Angina" or "Pectoris" or "Stenocardia" or "Stenocardias" or "Angor Pectoris" 14830

#6 #4 or #5 14830

#7 MeSH descriptor: [Coronary Artery Disease] explode all trees 7112

#8 "Artery Disease, Coronary" or "Artery Diseases, Coronary" or "Coronary Artery Diseases" or "Left Main Coronary Artery Disease" or "Left Main Disease" or "Left Main Diseases" or "Left Main Coronary Disease" or "Coronary Arteriosclerosis" or "Arterioscleroses, Coronary" or "Coronary Arterioscleroses" or "Atherosclerosis, Coronary" or "Atheroscleroses, Coronary" or "Coronary Atheroscleroses" or "Coronary Atherosclerosis" or "Arteriosclerosis, Coronary" 4952

#9 #7 or #8 8604

#10 MeSH descriptor: [Cardiovascular Diseases] explode all trees 116691

#11 "Diseases, Cardiovascular" or "Disease, Cardiovascular" or "Cardiovascular Disease" 25598

#12 #10 or #11 135389

#13 #3 and #6 34

#14 #3 and #9 7

#15 #3 and #12 49

**4. Search strategy for Wanfang**

检索表达式（主题词扩展）： 主题:(震波治疗) and 主题:(心脏)
