# Supplementary material for: Cardiac Shock Wave Therapy in Coronary Artery Disease: A Systematic Review and Meta-Analysis
Source: Front Cardiovasc Med. 2022 Jul 25;9:932193. doi: 10.3389/fcvm.2022.932193 (PMC9358011; doi:10.3389/fcvm.2022.932193)
Supplement: Supplementary file 2 [file Table_2.DOCX]

**Assessment results of risk of bias by RoB 2**

|  | **Randomization process** | **Deviations from intended interventions** | **Mising outcome data** | **Measurement of the outcome** | **Selection of the reported result** | **Overall Bias** |
| --- | --- | --- | --- | --- | --- | --- |
| **Study ID** |  |  |  |  |  |  |
|  |  |  |  |  |  |  |
| Čelutkienė | Low | Low | Low | Low | Low | Low |
| WJ Liu | Low | Low | Low | Low | Low | Low |
| N Jia | Low | Low | Low | Low | Low | Low |
| P Yang | Low | Low | Low | Some concerns | Low | Some concerns |
| Y Wang | Low | Low | Low | Some concerns | Low | Some concerns |
| L Ma | Some concerns | Low | Low | Low | Low | Some concerns |
| MX Song | Some concerns | Low | Low | Low | Low | Some concerns |
| L Zhang | Some concerns | Low | Low | Low | Low | Some concerns |

**Assessment results of risk of bias by ROBINS-tool**

| **Study** | **Confounding** | **Selection of participants into the study** | **Classification**  **of interventions** | **Deviations from intended interventions** | **Missing data** | **Measurement of**  **outcomes** | **Selection of the reported result** | **Overall Bias** |
| --- | --- | --- | --- | --- | --- | --- | --- | --- |
| **Kagaya** | moderate | moderate | low | low | low | low | moderate | moderate |
| **Alunni** | low | low | low | low | low | low | low | low |
